# Predictability of Fall Risk Assessments in Community-Dwelling Older Adults: A Scoping Review

**DOI:** 10.3390/s23187686

**Published:** 2023-09-06

**Authors:** N. F. J. Waterval, C. M. Claassen, F. C. T. van der Helm, E. van der Kruk

**Affiliations:** 1 Department of Rehabilitation Medicine, Amsterdam UMC Location University of Amsterdam, Meibergdreef 9, 1105 AZ Amsterdam, The Netherlands; n.f.waterval@amsterdamumc.nl; 2 Amsterdam Movement Sciences, Rehabilitation and Development, Amsterdam, The Netherlands; 3 Biomechatronics & Human-Machine Control, Department of Biomechanical Engineering, Faculty of Mechanical Engineering (3me), Delft University of Technology, Mekelweg 2, 2628 CD Delft, The Netherlands

**Keywords:** fall risk assessment, aging population, community dwelling older adults, sensor

## Abstract

Fall risk increases with age, and one-third of adults over 65 years old experience a fall annually. Due to the aging population, the number of falls and related medical costs will progressively increase. Correct prediction of who will fall in the future is necessary to timely intervene in order to prevent falls. Therefore, the aim of this scoping review is to determine the predictive value of fall risk assessments in community-dwelling older adults using prospective studies. A total of 37 studies were included that evaluated clinical assessments (questionnaires, physical assessments, or a combination), sensor-based clinical assessments, or sensor- based daily life assessments using prospective study designs. The posttest probability of falling or not falling was calculated. In general, fallers were better classified than non-fallers. Questionnaires had a lower predictive capability compared to the other assessment types. Contrary to conclusions drawn in reviews that include retrospective studies, the predictive value of physical tests evaluated in prospective studies varies largely, with only smaller-sampled studies showing good predictive capabilities. Sensor-based fall risk assessments are promising and improve with task complexity, although they have only been evaluated in relatively small samples. In conclusion, fall risk prediction using sensor data seems to outperform conventional tests, but the method’s validity needs to be confirmed by large prospective studies.

## 1. Introduction

One-third of people above 65 years of age experience a fall annually, and in more than 50% of the cases, medical assistance is needed [1,2,3]. In addition to direct injuries, older adults who experience a fall often suffer from fear of falling [4], reduced activity [5,6], and a lower quality of life [5]. Due to the rapidly aging population, fall prevalence and associated medical costs will progressively increase in the next decades.

The risk of falling increases due to extrinsic and intrinsic factors [7,8,9]. Extrinsic factors are external to the individual and include poor lighting, unsafe stairs, slippery floors, a loose carpet, or unsafe footwear [7,10]. Intrinsic factors can be divided into age-related physiological changes, pathological predisposing factors [9], and drugs [11]. To maintain balance, multiple physiological systems need to work in synergy, such as the sensory system, central nervous system, and motor system [12]. Most falls in older adults are a consequence of age-related reduction in the capacity of these neuromuscular systems [11].

Identification of older adults with increased risk of falling due to intrinsic factors is important to enable timely prescription of fall prevention programs and assistive devices, [13,14]. For this purpose, clinical fall risk assessments have been used for decades and include questionnaires (e.g., the Falls Efficacy Scale-international (FES-i) [15] and the Activity-specific Balance Confidence Scale (ABC-scale) [16]) or physical tests (e.g., the Tinetti Performance-Oriented Mobility Assessment (POMA) [17] and the Timed Up and Go test (TUG) [18]).

In the last decade, sensor-based fall risk assessments have gained more interest, as they have become more easily accessible by using wearable or portable sensors, provide more data, and may be a less time-consuming approach [19,20]. Sensor data can be collected during clinical fall-risk assessments [21,22], or during daily life activities [23,24].

Although fall prediction with sensor-based data is promising, recent reviews conclude that such predictions are often validated against fall risk classification based on clinical tests or fall history [25,26,27,28,29]. As sensor-based data are noisy, and signals of older adults may be even more complex, external validation using prospective study designs is especially important with sensor-based approaches in this population. Hence, reviews analyzing both prospective and retrospective fallers may not accurately describe the predictive capability of sensor-based approaches for future falls. Furthermore, currently, no overview of the predictive value of both clinical tests and sensor-based approaches exists, which makes it difficult to put the predictive value of sensor-based approaches in perspective [30]. Therefore, in this scoping review an overview of the predictive capability of clinical and sensor-based fall risk assessment for future falls in community-dwelling older adults is provided.

## 2. Methods

### 2.1. Search Strategy

In December 2021 the databases of Scopus, PubMed, IEEE Xplore, and Web of Science were searched using the following search string: (fall risk predict* OR fall risk assess* OR fall risk classif* OR fall risk measur*) AND (”older adult” OR aged OR elder* OR senior* OR geriatric) in the title, abstract, or keywords, and all fields were searched for (accura* OR sensitiv* OR specific*). An update of the search was performed in July 2023. Results were exported to EndNote (EndNote X9.3.3, Philadelphia, PA, USA) for further analysis.

### 2.2. Selection Criteria

Duplicates, conference proceedings, books, and serials were removed from the results. The search included 57 high-quality review articles on fall prediction published between 1988 and 2020. Therefore, the selection of high-quality studies was performed by one author (CK) searching the references from these recently published reviews using the following inclusion criteria: (i) inclusion of community-dwelling people with an age >60, or the mean age minus the standard deviation being 60; (ii) use of prospective methods for the categorization of fallers/non-fallers or low/high fall risk to avoid recall bias [31,32]; (iii) data are prescribed (or could be determined) on the number of fallers who were positive or negative for a fall risk assessment or summary statistics (sensitivity, specificity, or area under the curve (AUC)); and (iv) written in English or Dutch. Studies were excluded if (i) assistance by another person was allowed during the assessment, or (ii) the focus was only on the detection of (near) falls instead of predicting falls.

### 2.3. Data Extraction

Articles were categorized based on the type of fall risk assessment: clinical (without sensors) or sensor-based (during clinical or ADL assessments). The following data were extracted: number of participants, number of fallers and fall criteria, follow-up time, percentage female, mean age (SD), and sensitivity/specificity or positive/negative likelihood ratio.

For clinical assessments, studies that reported a predictive value in multivariate analysis and that reported validity measures were included. The specific assessment and cut-off score were extracted. For the sensor-based assessments, sensor type, sensor location, type of assessment, cut-off score, and validity measures were extracted.

### 2.4. Analysis

To compare results, we calculated the posttest probability (PoTP) for each study based on the sensitivity/specificity or positive/negative likelihood ratio. The PoTP defines how much fall risk has shifted compared to the pretest probability (PrTP) [31]. For example, falls have a prevalence of 30% in the population of older adults, so the chance of falling is 30%. If a fall risk assessment has a +PoTP of 60%, then a person with a positive assessment has a 60% chance of falling, while the PrTP was 30%. On the other hand, if the −PoTP is 20%, then the chance someone with a negative assessment will fall reduces from 30% to 20%. Ideally, an assessment would have a high +PoTP and a low −PoTP, which indicates that people with a positive assessment experience a fall, and those with a negative assessment do not fall. The PrTP in this study is set at 30%, based on the fall risk of older adults in the general population [1,2,33]. The PoTP is calculated based on the available measures [33]. The +PoTP is calculated as: true positives/(true positives + false positives). The −PoTP is calculated as: 1 − (false negatives/(false negatives + true negatives)).

## 3. Results

### 3.1. Study Selection

**Figure 1:**
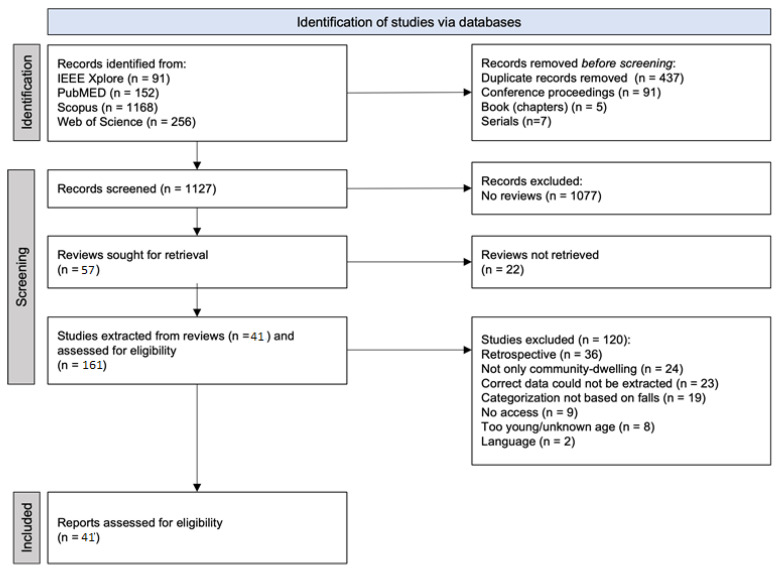
Flowchart of study selection.

**Figure 2:**
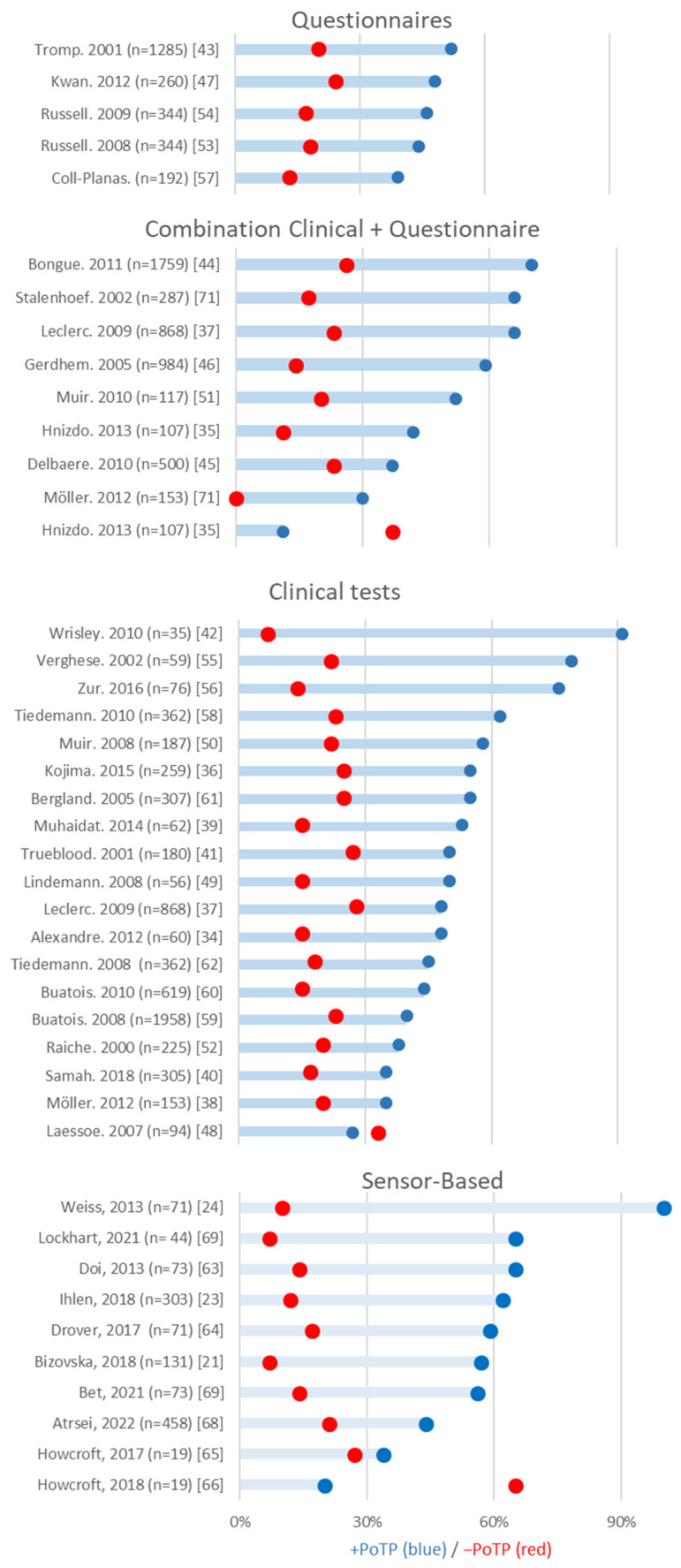
Predictive value of assessments sorted by +PoTP value. Only the best +PoTP per study is presented.

The combination of the results from IEEE Xplore (n = 91), PubMed (n = 152), Scopus (n = 1168), and Web of Science (n = 256) resulted in 1667 articles. After removing 437 duplicates, 1230 articles were left, of which 1127 were journal articles. After scanning the titles and abstracts, 57 reviews were included for full-text screening, from which 178 articles were extracted. After the full-text screening of these articles, 41 prospective studies were included. In Figure 1 the flow chart of the study selection has been displayed. Thirty articles focused on clinical assessments, based on questionnaires or physical tests, while two focused on sensor-based ADL assessments, and eight focused on sensor-based clinical assessments. The results regarding the +PoTP and −PoTP are shown in Figure 2.

### 3.2. Clinical Assessments without Sensors

Thirty articles focused on clinical assessments without sensors and were published between 2000 and 2018. Study characteristics are displayed in the Supplementary Material. In total, the studies included 12,406 participants, of whom 3084 were classified as fallers based on prospective data. The follow-up period varied from six months to three years [34,35,36,37,38,39,40,41,42,43]. The most used fall criterion was “at least one fall during follow-up” [32,33,34,35,36,38,39,40,41,42,44,45,46,47,48,49,50,51,52,53,54,55,56]. The 30 studies used a total of 39 different clinical assessments without sensors. These assessments can be classified as questionnaires, questionnaires combined with physical performance, and only physical performance.

### 3.3. Questionnaires

The predictive value of questionnaires was evaluated by a total of five studies evaluating four questionnaires: the fall-risk screening test [43], the Fall Risk for Older People in the Community Screen (FROP-Com) [53,54], the Geriatric Depression Scale (GDS) [47], and the combination of the history of falls and independent bathing [57]. The best performance for classification of fallers (+PoTP) based on questionnaires was found for the fall-risk screening test (+PoTP: 52%) [43]. This test was evaluated in one study (*n* = 1285) with a 3-year follow-up. It demonstrated classification of non-fallers (−PoTP) of 20% [43]. The FROP-Com was evaluated in two studies with a 12-month follow-up and had a slightly lower +PoTP but a slightly better −PoTP: (+PoTP: 46%, −PoTP: 17%) (*n* = 344) [53] and (+PoTP: 44%, −PoTP: 18%) (*n* = 192) [54]. The +PoTP of the GDS was slightly better than that of the FROP-Com (48%), yet the −PoTP was worse (24%) (*n* = 260) [47]. The combination of the history of falls and independent bathing was studied by one study for one fall in 12 months and classified non-fallers (−PoTP: 13%) better than fallers (+PoTP: 39%) (*n* = 192) [57].

### 3.4. Physical Performance

Assessments that use a stand-alone physical performance test are the alternate step test [58], adjusted maximum step length [49], Berg Balance Scale (BBS) [37,50,56], Dynamic Gait Index (DGI) [42], Functional Gait Assessment (FGA) [42], five-times sit-to-stand test (FTSS) [59,60], Zur balance scale [56], getting up from lying on the floor [61], one-leg balance (OLB) [60], Tinetti Performance-Oriented Mobility Assessment (POMA) [21,41,52,55], risk assessment [60], stair ascent [62], test battery [48], timed gait [55,62], TUG [34,36,37,38,39,40,41,42,59], and walking while talking test (WWT) [55]. The TUG had the highest predictive value, but the +PoTP varied from 31% to 91% and the −PoTP from 7% to 29% between the nine articles, all but one of which had a follow-up of 6 months (Supplementary Material Table S1). The best scores (+PoTP: 91%, −PoTP: 7%) are from a small study (*n* = 35), where the average of three TUG tests was used [42]. The next best results for the TUG had +PoTPs of 55% and 48% and −PoTPs of 25% and 15% using larger sample sizes (*n* = 259 and *n* = 60, respectively) [34,36]. The studies with the largest sample sizes for the TUG evaluation (*n* = 621 and n = 868) (2 falls) had even lower +PoTP values of 42% and 37%, and −PoTP values of 29% and 28%, respectively [37,60].

The second-best predictor of fall risk in fallers with a physical test only was the WWT (complex: +PoTP: 79%, −PoTP: 22%; simple:+PoTP: 65%, −PoTP: 21%) [55]. This test was however evaluated in a single prospective study with a relatively small sample size (*n* = 59), and the results should therefore be interpreted with caution. These same limitations (n = 94) hold for the studies on the Zur balance scale (+PoTP: 74%, −PoTP: 16%) [56], FGA (FGA) (+PoTP: 71%, −PoTP: 0.00%) [42], the DGI (+PoTP: 64%, −PoTP: 0.00%) [42], the classification tree (+PoTP: 53%, −PoTP: 15%) [39], and the test battery (+PoTP: 27%, −PoTP: 33%) [48]. The POMA was verified in four studies, but in distinct forms: the complete short assessment (full POMA) [21,41], the balance part of the short assessment only (14-item balance assessments) [21,55], and the balance part of the long assessment only (nine-task balance part) [52]. The different forms have comparable posttest probabilities: The full POMA (n = 180 +PoTP: 50%, −PoTP: 27% [41], n = 131 +PoTP: 63%, −PoTP: 15% [21]), 14-item balance assessments (n = 225 +PoTP: 38%,−PoTP: 20% [55], n = 131 +PoTP: 65%, −PoTP: 20% [21]) and the nine-task balance part (n = 59 +PoTP: 48%,−PoTP: 18%) [52].

The risk model for recurrent falls [61], performance-based FRAT (6) (+PoTP: 62%, −PoTP: 23%) (n = 362) [58], and getting up from lying on the floor (+PoTP: 55%, −PoTP: 25%) (n = 307) [62] demonstrated good predictive value in large cohort studies. Tiedemann et al. (2010) compared several cut-off scores for the performance-based FRAT (0–1, 2–3, 4–5, 6) and reported the best posttest probabilities with a cut-off of 6 [58]. Timed gait was used in different tasks. In the study of Verghese et al. (2002) (*n* = 59), the duration of a participant walking 6 m, turning, and returning at a normal walking speed was measured [55], while Tiedemann et al. (2008) (*n* = 347) timed a 6 m straight walk at a normal pace [63]. The timed gait with turn showed a slightly better predictive value (+PoTP: 52%, −PoTP: 24%) [52] than the test without the turn (+PoTP: 40%, −PoTP: 24%) [60].

### 3.5. Sensor-Based Clinical Assessments

The performance of identification of fallers using sensor-based clinical assessment is dependent on the task, the sensor location, feature extraction, and the classification method. The tasks used in the prospective sensor-based studies were standardized walking tests [21,64,65,66,67,68], of which five only used data from straight walking, one used straight walking and turns during a 6 min walk test (6MWT) [64], one used a five-times sit-to-stand test [69], and several used TUG tests [70] (Table 1). Sensors were worn on various locations of the body, namely the sternum (2×), at L3 on the back (2×), and on the lower back in combination with a shank (4×), of which two studies also placed an accelerometer on the head. Only the study of Atrsaei classified more than 50 people as fallers [69]. 

The study of Drover et al. analyzing both straight walking and turning concluded that analyzing turning (+PoTP: 59%, −PoTP: 17%) and turning and straight walking (+PoTP: 58%, −PoTP: 18%) had a higher predictive value than straight walking alone (+PoTP: 33%, −PoTP: 26%) [65]. For analysis, the maximum, mean, and SD of acceleration in all directions of the three axes, acceleration frequency, and ratio of even/odd harmonics from sensors on the shank were used in combination with a random forest classifier. That turns are important is in agreement with the results are of Bet et al. [70], who used the TUG test, which also includes a turn (+PoTP: 56%, −PoTP: 14%). On the contrary, Artsaei et al. [69], which used a sit-to-stand test, was by far the largest study using a sensor-based approach (n = 458) and had a lower predictive value (+PoTP: 44%, −PoTP: 21%). Howcroft, Koftmann, and Lemaire (2017 and 2018) compared sensor locations for optimal classification of prospective falls [66,67]. They analyzed single-task (ST) and dual-task (DT) walking with sensors on the head, pelvis, ankles, and with a pressure insole [66,67] using a vector machine (SVM) and neural networks. Based on their prospective study results, they conclude that in DT the pelvis accelerometer had the best single-sensor predictive capability, while in ST the head location performed better. Overall, their conclusion was that multi-location sensors outperformed the single-sensor approach. Bizovska et al. (2018) used sensors on the trunk and shanks and found that the sensors on the shanks did not contribute to a distinction between fallers and non-fallers [21]. In this study the trunk medial–lateral (ML) acceleration in the short term (slopes of mean log divergence curve between 0 and 0.5 stride) and Lyapunov exponents (stLE) had the best predictive power during a 25 m straight walk (+PoTP: 60%, −PoTP: 19%). Doi et al. found that the harmonic rate in the vertical direction of the sensor on the upper trunk was the discriminating factor for a 15 m straight walk (+PoTP: 65%, −PoTP: 14%) [64].

### 3.6. Sensor-Based ADL Assessments

Two studies used sensors in daily living, one for three days [24] and one for a week [23]. Both studies used a fall criterion of two falls in 6 months and made use of accelerometer data from the lower back (lumbar spine). Weiss et al. combined the 3-day measurements with the Dynamic Gait Index (DGI) in 71 participants, of whom 12 were fallers. Fallers and non-fallers could be classified by the total activity duration, DGI, and the anterior–posterior acceleration range and width, extracted from the frequency in the power spectral density [24]. The combination of DGI and 3-day ADL had a sublime result of a +PoTP of 100% and a −PoTP of 10% [24]. Ihlen et al. used phase-dependent generalized multiscale entropy (PGME) to define time series irregularities in 303 participants. They investigated the high-frequency intra-step modulation of trunk acceleration signals of walking [23] and found a +PoTP of 74% and a −PoTP of 14% [23]. Combining the fall history, conventional gait, and demographic variables with the sensor data resulted in a worse +PoTP (64%) and a better −PoTP (8%).

## 4. Discussion

The aim of this scoping review was to provide an overview of the predictive capability of clinical fall assessment tools and sensor-based approaches in community-dwelling older adults. In general, all assessment tools classified fallers better than non-fallers. Across the different clinically used tools, questionnaires had a lower predictive value compared to physical tests. Sensor-based assessment seems to outperform clinical tests when using complex tasks such as turning, although the reliability of these results is limited due to the studies’ small sample sizes.

In this scoping review, we defined the predictive capability using the posttest probability, where a higher +PoTP means falls can be more accurately predicted. As approximately 30% of older adults fall each year [1,2,33], we define a cut-off above a correct prediction of fallers of 55%. This means 25% more falls are predicted than could be expected based on chance. Questionnaires had a lower predictive capability than the other assessment types, as no study evaluating questionnaires found a +PoTP value above 52%. The predictive value was improved by combining questionnaires with physical performance tests and/or including specific questions about fall history or medication use. However, taking fall history into account has its limitations, as it cannot be used to predict who is at risk of falling for the first time. Therefore, although easy to administer, the usability of questionnaires to predict future falls is currently limited.

Physical clinical fall assessment tools such as TUG, BBS, and FTSS have been evaluated by multiple studies but do not show high predictive capability for prospective falls in larger trials. All studies using the TUG, except for one (+PoTP of 91%) which only included six fallers [42], showed a +PoTP of 55% or lower, indicating that falls cannot be accurately predicted. The same holds true for the FTSS and BBS, where only a relatively small-sampled study [54] was promising, while the larger studies showed insufficient predictive capability, with +PoTP scores of <48% [37,50]. Newer clinical tests such as the risk model for recurrent falls, performance-based FRAT, and getting up from lying on the floor showed promising predictive values (+PoTP > 55%) in studies with moderate sample sizes (N between 145–303). However, given that larger studies tend to have a lower predictive capability, and that the predictive value depends largely on the cut-off score used [58], it is too early to conclude that these tests perform better. The relatively low predictive capability of physical tests is in contrast with the review of Lusardi et al. (2017), who concluded that the BBS, TUG, and FTSS are currently the most evidence-supported functional measures to determine individual fall risk. However, this review included retrospective studies [33], for which much better associations between TUG, BBS, and FTSS and falls have been reported, as demonstrated by Beauchet et al. (2011) [30].

Sensor-based assessments demonstrate promising predictive capability in relatively small samples, although the predictive capability depends on the task complexity, the location of the sensor, and feature extraction. The current studies mainly used 3D accelerometers placed on the back or sternum and analyzed sensor data of standardized tests, both due to convenience. Potentially, other sensor locations, such as the head, are more suitable to detect changes in movement, as human motor control aims to stabilize head positioning. This is supported by the findings of Howcraft et al. [65,66,67], where the head was the best predictor during straight walking. However, during a double task, the pelvis sensor performed better, indicating that the best location may be task-specific. Furthermore, even with a head sensor, straight walking does not yield enough discriminative information to predict fallers, as the +PoTP was below 40%. More complex tasks such as turning during walking [65], multiple TUG tests [70], or dual-task walking [66] showed better predictability, which is also in line with the previous review of Bayot et al. (2020) [71]. This indicates that to be able to discriminate future fallers from non-fallers, more complex tasks than straight walking need to be performed.

A potential advantage of sensor-based approaches is the ability to conduct measurements in daily life, where complex tasks are performed constantly. In our scope, we identified only two studies collecting sensor data in daily life, both showing good predictive capability (+PoTP > 74%) [23,24]. The sensor was placed on the lower back in both studies, which might not be the most sensitive location for detection of movement alterations. A disadvantage of measuring in daily life is the complexity of data processing due to the variation in tasks and environments. Consequently, collecting sensor data during a complex standardized task might be more easily implemented.

## 5. Limitations and Future Work

In this work, we aimed to provide an overview of the existing evidence of both traditional clinical fall assessment tools and sensor-based approaches. By using a scoping method, we are confident that we have included the majority of the relevant literature, although, as no systematic approach was used, we may have missed relevant studies. A strength is the inclusion of only prospective studies, as inclusion of studies classifying fallers based on retrospective falls can lead to an overestimation of the predictive value of a method. Furthermore, we recalculated the fall prediction of each study to a posttest probability, meaning direct comparison between studies was possible. Our scope suggests that sensor-based fall risk assessment potentially outperforms traditional assessments, which warrants large, high-quality prospective studies to assure the reliability of the sensor-based approaches. Future research should aim to determine the best location of the sensors and the proper task to perform with the sensors in use.

## 6. Conclusions

The use of sensors during clinical tests or daily life has the potential to improve prediction of future falls compared to standardized clinical tests. However, large prospective studies to better determine the predictive capability and select the most suitable sensor location and tasks are warranted to fulfill this potential.

## Figures and Tables

**Table 1:** Overview study characteristics and results of sensor-based assessments.

Author	Total (n)	Female (%)	Mean Age (SD)	Fallers	Fall Criteria	Follow-Up Time (Months)	Type of Sensor	Sensor Position	Assessment	Analyzed		+PoTP	−PoTP
Atrsaei, 2021 [69]	458	57	74.9 (1.4)	108	>=2 or >=1 injury due to fall	12, fall calendar report monthly	1 3D accelerometer 1 3D gyroscope	Sternum	5xSTS			44%	21%
Bet, 2022 [70]	73	56	70.2 (6.7)	15	>=1	12, fall journal contacted every 3 months	1 3D accelerometer	Waist, L3	TUG, TUG-DT			56%	14%
Bizovska, 2018 [21]	131	NR	NF: 70.5 (6.4) MF: 71.2 (5.3)	SF: 35 MF: 15	>=2	12, every 14 days called to report	3 3D accelerometers	Trunk (near L5) Left and right shank (15 cm above malleolus)	25 m walking	Trunk stLE ML		60%	19%
										Tinetti balance score, trunk stLE ML		47%	20%
										Tinetti total score, trunk stLE ML		57%	7%
										Tinetti balance score, Tinetti total score, trunk stLE ML		55%	11%
Doi, 2013 [64]	73	78.1	80.3	SF: 16	>=1	12, self-reporting weekly collection	2 3D accelerometers	Upper trunk (C7) Lower trunk (L3)	15 m walking			65%	14%
Drover, 2017 [65]	71	NR	74.15 (7.0)	SF: 28	>=1	6, fall occurrence survey after 6 months	3 accelerometers	Lower back Left and right lateral shank Acc: posterior head	6MWT			57%	18%
Howcroft, 2017 [66]	19	58.7	75.2 (6.6)	SF: 7	>=1	6, fall calendar report monthly	1 accelerometer, 1 pressure sensor	Lower back Lateral shank just above the ankle Pressure insole: plantar H-RS H-P-LS Acc: posterior head	7.62 m walking (ST, DT and 6MWT (ST)	single-task walking dual-task walking		34%	27%
										single-task walking		33%	28%
										dual-task walking		36%	27%
Howcroft, 2018 [67]	19	58.7	75.2 (6.6)	SF: 7	>=1	6, fall calendar report monthly	1 accelerometer, 1 pressure sensor	Lower back Lateral shank just above the ankle Pressure insole: plantar	ST Walking			20%	65%
Ihlen, 2018 [23]	303	SF: 51 ME: 48.8	SF:76 (6.8) MF: 75.9 (6.7)	SF: 58 MF: 46	>=1 >=2	12, monthly phone calls	1 3D accelerometer	Lower back	1-week ADL		PGME	60%	13%
											Conventional gait and demographic variables	52%	18%
											Fall history	38%	25%
											All combined	62%	12%
											PGME	74%	14%
											Conventional gait and demographic variables	59%	11%
											Fall history	40%	23%
Lockhart 2021 [68]	44	NR	73.0 (8.0)	SF: 9	>=1	6, self-report	1 3D accelerometer	Sternum	10 m walk			65%	7%
Weiss, 2013 [24]	71	65	78.36 (4.71)	MF: 12	>=2	6	1 3D accelerometer 1 3D gyroscope	Lower back	3-day ADL	All combined		64%	8%
										Dynamic gait index (without sensors)		87%	23%
										DGI (without sensors) + 3-day acceleration-derived		100%	10%

6MWT: 6 min walk test, Accel: accelerometer, ADL: activities of daily living, DGI: Dynamic Gait Index, DT: dual-task, INF: infinity, MF: multi-faller, PGME: phase-dependent generalized multiscale entropy, SF: single faller, ST: single-task, stLE ML: short-term Lyapunox exponents, TUG: Timed-Up and Go. +PoTP = positive posttest probability. −PoTP = negative posttest probability.

## Data Availability

Not applicable.

## Supplementary Materials

The following supporting information can be downloaded at: https://www.mdpi.com/article/10.3390/s23187686/s1. Table S1 with study characteristics of clinical assessments.
