# Supplementary material for: Predictability of Fall Risk Assessments in Community-Dwelling Older Adults: A Scoping Review
Source: Sensors (Basel). 2023 Sep 6;23(18):7686. doi: 10.3390/s23187686 (PMC10536675; doi:10.3390/s23187686)
Supplement: Supplementary file 1 [file sensors-23-07686-s001.zip › sensors-2444910-supplementary.pdf]

Supplementary material

Table 1: Study characteristics of clinical assessments.

Author, year	Total (n)	Female (%)	Mean age (SD)	Fallers	Fall criteria	Follow-up time (months)	Tool
**Questionnaires**							
Coll-Planas, 2006	192			SF: 116	IFs	12, self-report	History of falls & independent bathing/showering
Kwan, 2012	260	30.5	74.9 (6.4)	SF: 51, MF: 35	>=1<br>>=2	24, montlhy telephone contact	GDS
Russell, 2008	344	69.2	75.9 (8.5)	SF: 64, MF: 100	>=1	12, falls diary, reported every 2 months by phone	FROP-com
Russell, 2009	344<br>263	69.2	75.9		>=1<br>>=2	12, fall diaries	FROP-Com
Samah, 2018	305	43.9	67.67 (5.5)	SF: 40	>=1	6, falls diary, monthly report by phone call	TUG
Tiedemann, 2008	362	NR	80.4 (4.5)	SF: 99, MF: 80	>= 2	12, montlhy fall calendars	Alternate step test<br>FTSS<br>Timed gait (6 m)Stair ascent
Tromp, 2001	1285	51	75.2 (6.5)	MF: 146	>=2	36, weekly report of falls onfall calendar, mail calendarevery 3 months	Fall-risk screening test
Trueblood, 2001	180	79.4	77.9 (7.26)	SF: 30, of whom IF: 16	>=1	6, report via phone at4 and 6 months	POMA<br>TUG<br>Reaction time etc.*
Verghese, 2002	59	57.6	79.6 (6.3)	SF: 13	>=1	12, phone interview at6 and 12 months	POMA, gait only<br>Timed Gait WWT-simple WWT-complex
Wrisley, 2010	35	51.4	72.9 (7.8)	SF: 6	>=1	6, postage-paid fall calendar postcards report monthly	DGI<br>FGA<br>TUG<br>BBS
Zur, 2016	76	79	83 (5)	SF: 8, MF: 5	>=1	18, from medical records	Zur balance scale
**Physical Test**							
Alexandre, 2012	60	51.7	66	SF: 19	>=1	6, self report of a fall, reportedafter 3, 6 and 12 months	TUG
Bergland, 2005	307	100	80.8 (range 75-93)	SF: 61, IF	Severe IF	12, fall calendar, report every 3 months	Getting up from lying on the floor
Bizovska, 2018	131	SF: 35, MF: 15	NR	NF: 70.5 (6.4), M: 71.2 (5.3)	>=2	12, every 14 days called to report	16-item full POMA
Buatois, 2008	1958	50	70 (4)	SF: 239, MF: 183	>=2	18-36, questionnaire	POMA balance<br>FTSS
Buatois, 2010	619	50	70.1 (4.4)	MF: 55	>=2	25, questionnaire	FTSS<br>TUG<br>OLB

Table 1 continued from previous page

Author, year	Total (n)	Female (%)	Mean age (SD)	Fallers	Fall criteria	Follow-up time (months)	Tool
Kojima, 2015	259	63.3	72.6 (5.9)	38, MF: 21	>=1	6, fall diaries, monthly report by mail	Risk assessmentSF: TUG
Laessoe, 2007	94	74	73.7 (2.9)	SF: 14	>=1	12, fall diary, report by phoneevery 6 and 12 months	Test battery
LeClerc, 2009	868	77.2	F: 79.5 (6.6) NF: 79.0 (6.9)	MF: 99	>=2	6, calendar with monthlyreport by telephone	Classification tree for risk of recurrent falling
							BBS
							TUG
Lindemann, 2008	56	57	67.7 (6.0)	SF: 30	>=1	12, daily fall calendar, every 2 months called, send calendarsafter 12 months	Step length assessment AmeanVL AmaxVL AmeanVL + history of falls (12 months)AmaxVL + history of falls (12 months)
				SF: 38 of whom 68.4% IF	>=1	6, question about fallsin past 3 months	TUG
						12	TUG
						6	DFRI
Moller, 2012	153	67	81.5 (6.3)			12	DFRI
Muhaidat, 2014	62	NF:65.3 F:69.2	NF: 75 (11.5) F: 82 (12)	SF: 13	>=1	6, fall diary, reporteach months by post	Classification tree
Muir, 2008	187	35	79.47 (5.83)	SF: 80 of whom MF: 33, IF: 55	>=1	12, daily registration in fall calendar, monthly report by mail	BBS
					>=2		
					IFs		
Raiche, 2000	225	NR	80.0 (4.4)	SF: 53	>=1	12, calendar to record the date of any falls, monthly report by phone	14-item POMA
**Questionnaires & Physical Test**							
Bongue, 2011	1759	51	70.7 (4.6)	SF: 563	>=1	12, monthy collected by phone	Clinical screening tool
Delbaere, 2010	500	54	77.9 (4.6)		>=1 IF, >=2 falls	12, fall diary, monthly report	PPA
							PPA followed by FES-I
Gerdhem, 2005	984	100	75 (range: 75.01-75.99)	MF: 232	>=1	12, questionnaire after 12 months	mJH-FRAT
Hnidzo, 2013	107	34.6	79.8	SF: 33, IF: 7	>=1	6, daily fall calendar, report each month	mJH-FRAT
					IFs		

Table 1 continued from previous page

Author, year	Total (n)	Female (%)	Mean age (SD)	Fallers	Fall criteria	Follow-up time (months)	Tool
LeClerc, 2009	868	77.2	F: 79.5 (6.6)<br>NF: 79.0 (6.9)	MF: 99	>=2	6, calendar with monthly report by telephone	Classification tree for risk of recurrent falling<br>BBS<br>TUG
Moller, 2012	153	67	81.5 (6.3)	SF: 38 of whom 68.4% IF	>=1	6, question about falls in past 3 months	TUG
						12	TUG
						6	DFRI
						12	DFRI
Muir, 2010	117		79.7 (5.3)	SF: 26, MF: 26, IF: 36	>=1<br>IF	12, daily falls calendar, monthly submission	AGS/BGS/AAOS<br>Fall screening algorithm
Stalenhoef, 2002	287	19.5	Female 78.5 (5.2)<br>Male 77.2 (4.9)	SF: 49, MF:46	>=2	9, 6-weekly report by telephone	Risk Model for recurrent falls
Tiedemann, 2010	362	78.7	75.3 (5.8)	MF: 80	>=2	12, monthly fall calendars	Performance-based FRAT
	362	65	80.25 (4.5)	MF: 80		12	Performance-based FRAT

Abbreviations: AGS/BGS/AAOS: American Geriatrics Society/British Geriatrics Society/American College of Orthopaedic Surgeons, AmaxVL: adjusted maximum valid step length, AmeanVL: adjusted mean valid step length, BBS: Berg Balance Scale, DFRI: Downton Fall Risk Index, F: faller, FES-I: fall efficacy scale international, FGA: functional gait assessment, FROP-Com: fall risk of older people in the community, FTSS: Five-times sit-to-stand, GDS: geriatric depression scale, IF: injurious falls/fallers, mJH-FRAT MF: multi-falls/fallers, NF: Non-faller, OLB: one-leg balance test, POMA: Tinetti Performance Oriented Mobility Assessment, PPA: physical performance assessment, SF: single faller, TUG: Timed Up and Go test, WWT: walking while talking.

*Age, fal history, reaction time, movement velocity, standing on a firm plate with eyes open
